# Editorial: Renal Hypertension at the Crossroads: Theoretical, Experimental and Clinical Aspects

**DOI:** 10.3389/fmed.2020.00049

**Published:** 2020-02-19

**Authors:** Maik Gollasch, Joachim Hoyer

**Affiliations:** ^1^Nephrology/Intensive Care, Charité Medical University of Berlin, Berlin, Germany; ^2^Department of Internal Medicine and Geriatrics, University of Greifswald, Greifswald, Germany; ^3^Experimental and Clinical Research Center, Charite University Medicine Berlin, Berlin, Germany; ^4^Department of Internal Medicine and Nephrology, University Medicine Marburg, Marburg, Germany

**Keywords:** hypertension, perivascular adipose tissue, renal denervation, caveolae, alamandine

Since Goldblatt's seminal experiment in Goldblatt et al. (1), renal hypertension has increasingly been recognized as an important cause of secondary hypertension and chronic kidney disease, the latter by virtue of renal ischemia. Followed later by important work, the scientific community recognized that renal artery occlusion does not only increase systemic blood pressure by decreased transstenotic blood flow but by an array of multiple systemic or local mechanisms, which all contribute to high blood pressure and hypertension-related injury. The contributions made in this Frontiers Research Topic are significant because they add to considerations that have been given to the kidney in hypertension and relative to its role in vascular function. The control of renal and cardiovascular hemodynamics and renal function in hypertension was elegantly discussed (Sata et al.). Resistant hypertension is generally defined as uncontrolled blood pressure (>140/90 mm Hg) after treatment with three or more antihypertensives. Salt sensitivity was studied in resistant hypertension and renal denervation using an *in vitro* erythrocyte salt sedimentation assay (Vonend et al.). Alamandine and its receptor were identified as novel protective contributors to the renin-angiotensin system (Schleifenbaum). A novel hierarchical statistical method and genetically encoded calcium indicators (GECIs) have been implemented in experimental hypertension (Tsvetkov et al.; Zhong and Schleifenbaum). Caveolae associated signal transduction pathways were identified in vasculature to play important pathophysiological role in hypertension (Lian et al.). Together, the authors explicitly place findings that can be taken advantage of in creating new therapies and experimental models for cardiovascular diseases that continue to challenge our community. These include resistant hypertension, renal artery interventions, therapeutic targeting of endothelial cells and perivascular adipose tissue, and salt consumption in health and disease to name a few. We are grateful to our contributors for sharing their important work.

## Author Contributions

All authors listed have made a substantial, direct and intellectual contribution to the work, and approved it for publication.

### Conflict of Interest

The authors declare that the research was conducted in the absence of any commercial or financial relationships that could be construed as a potential conflict of interest.
